# Hamiltonian Monte Carlo methods for efficient parameter estimation in steady state dynamical systems

**DOI:** 10.1186/1471-2105-15-253

**Published:** 2014-07-28

**Authors:** Andrei Kramer, Ben Calderhead, Nicole Radde

**Affiliations:** Institute for Systems Theory and Automatic Control, Pfaffenwaldring 9, 70550 Stuttgart, Germany; Department of Mathematics, Imperial College London, London, SW7 2AZ UK

**Keywords:** MCMC methods, Parameter estimation, Hybrid monte carlo, Steady state data, Systems biology

## Abstract

**Background:**

Parameter estimation for differential equation models of intracellular processes is a highly relevant bu challenging task. The available experimental data do not usually contain enough information to identify all parameters uniquely, resulting in ill-posed estimation problems with often highly correlated parameters. Sampling-based Bayesian statistical approaches are appropriate for tackling this problem. The samples are typically generated via Markov chain Monte Carlo, however such methods are computationally expensive and their convergence may be slow, especially if there are strong correlations between parameters. Monte Carlo methods based on Euclidean or Riemannian Hamiltonian dynamics have been shown to outperform other samplers by making proposal moves that take the local sensitivities of the system’s states into account and accepting these moves with high probability. However, the high computational cost involved with calculating the Hamiltonian trajectories prevents their widespread use for all but the smallest differential equation models. The further development of efficient sampling algorithms is therefore an important step towards improving the statistical analysis of predictive models of intracellular processes.

**Results:**

We show how state of the art Hamiltonian Monte Carlo methods may be significantly improved for steady state dynamical models. We present a novel approach for efficiently calculating the required geometric quantities by tracking steady states across the Hamiltonian trajectories using a Newton-Raphson method and employing local sensitivity information. Using our approach, we compare both Euclidean and Riemannian versions of Hamiltonian Monte Carlo on three models for intracellular processes with real data and demonstrate at least an order of magnitude improvement in the effective sampling speed. We further demonstrate the wider applicability of our approach to other gradient based MCMC methods, such as those based on Langevin diffusions.

**Conclusion:**

Our approach is strictly benefitial in all test cases. The Matlab sources implementing our MCMC methodology is available from https://github.com/a-kramer/ode_rmhmc.

**Electronic supplementary material:**

The online version of this article (doi:10.1186/1471-2105-15-253) contains supplementary material, which is available to authorized users.

## Background

Parameter estimation is a major task that paves the way for building predictive models of intracellular regulation processes. Experimental data used for fitting these models however, often do not contain enough information to identify parameter values uniquely. Especially for parameter estimation of quantitative dynamic models such as ordinary differential equations (ODE) biological experiments usually do not have the required time resolution, rendering the estimation particularly challenging. Sparse data often lead to ill-posed optimization problems with multiple solutions that are indistinguishable in terms of fit quality, but might differ substantially when used for the prediction of new scenarios. Faced with these problems, optimization based point estimates are generally not appropriate, since an analysis of the parameter space around such points of high quality fits is often needed. Sampling-based Bayesian methods are advantageous in these settings as they offer an insight into the surroundings of local objective function minima and facilitate the uncertainty analysis of predictions. Consequently, statistical methods are being used more and more frequently for parameter estimation in systems biology (see e.g. [1, 2]). In the Bayesian approach, parameters are considered random variables and inferences are formulated in terms of probability densities.

A major hindrance is the time required to generate samples from the resulting posterior distributions. Evaluation of the posterior can be computationally expensive; this is particularly the case for ODE models, where each sampling step requires numerical calculation of model trajectories. Metropolis-Hastings [3, 4] algorithms are among the most popular choices for this task (see e.g. [2, 5]). Variants of these core algorithms are also widely available in parameter estimation software packages, e.g. GNU MCSIM[6], or the MCMCSTAT MATLAB toolbox [7].

The speed of convergence of the Markov chain is crucial for the efficiency of the MCMC approach. Finding a good tuning of the Markov chain’s transition distance can be time consuming and difficult [1, 8, 9], and it has been recognized that high correlations between the parameters can substantially slow down the convergence speed of standard MCMC algorithms [10]. In these cases the step size must often be chosen to be very small in order to obtain a reasonable acceptance rate, resulting in highly auto-correlated samples. A larger number of samples are then required to obtain low variance estimates of the inferred quantities.

Various strategies are employed to increase the distance of transitions in the sampling space, yet at the same time maintain a high acceptance rate. Several adaptive versions of the Metropolis-Hastings algorithm have been suggested in this context (see e.g. Ch.4 in [8]). These adaptation processes however only make proposals based on a global Gaussian approximation of the posterior, which can be a disadvantage when the posterior density has a complex lower scale structure.

Hybrid or Hamiltonian Monte Carlo (HMC) algorithms ([11]), can dramatically increase the acceptance rate while still providing samples with low auto-correlation. This is accomplished by an elaborate transition step that requires the integration of a secondary ODE system describing dynamics in parameter space that depend on the gradient of the target distribution. In this way, the HMC algorithm uses the structure of the ODE model more exhaustively. At first glance this approach increases the computational costs, but this is often compensated by the improved sample quality compared to the simpler algorithms. The advantage of low auto-correlations in typical HMC samples is that the sample size need not be as large to compute summaries to a satisfactory precision. A wasteful algorithm might provide a sample with high auto-correlations very quickly but at the expense of requiring a much larger number of samples to obtain low variance Monte Carlo estimates. Of course, these larger samples also need to be stored and empirical estimates of posterior predictive distributions using a larger number of sampled points will be computationally slower.

The statistical superiority of HMC has already been demonstrated on a variety of examples, e.g. using a 100-dimensional multivariate Gaussian distribution as target density (see Ch.5 in [8]); it seems to be a very promising approach, however HMC has so far rarely been used to infer parameters for nonlinear ODE models. A recent simple example is given in [1], where it is shown that the sample quality and convergence of HMC to the posterior can be improved even further by using an appropriate metric to define a Riemannian geometry over the parameter space. This approach employs the local sensitivity of the model at each step to inform the proposal distribution, leading to more efficient sampling in cases where the posterior has a complicated covariance structure.

The major difficulty that arises generally for HMC type algorithms when dealing with ODE models is that the model outputs and their sensitivities have to be simulated at every point along trajectories in parameter space. We address this computational issue by proposing an extension to HMC algorithms especially designed to sample efficiently from models with *steady state data* under multiple perturbations. In particular, we use sensitivity analysis within a Newton-Raphson approach to efficiently track the steady states across each of the Hamiltonian trajectories, instead of calculating them explicitly, thus drastically reducing this additional cost.

Steady state observations are typically not as informative as time series data, but can be obtained at comparatively low cost. They can be used in the first cycle of modeling where a qualitative model is validated via parameter fitting. If the model is not invalidated, dynamic data should be gathered for further analysis to narrow down model parameters further. The posterior parameter density from previous analysis cycles may be employed to inform the prior density of future experiments, and herein lies one of the benefits of the Bayesian approach. The properties of steady states offer the possibility to use analytical calculations to obtain output sensitivities. This is a lot faster than the numerical integration of dynamic sensitivities and improves sampling performance. We describe this further in Section ‘Efficient calculation of geometry’. Typically, the steady state data will not be sufficient to uniquely identify all parameters; however issues of unidentifiability may also occur for dynamic time series data. Bayesian model analysis is designed to deal with precisely this case and allows full characterisation of the uncertainties that arise when making predictions.

We evaluate our approach on three different steady state models with real data: a model for Erk phosphorylation in the MAPK signaling pathway, and two alternative models for the phosphorylation of insulin receptor substrate IRS after insulin stimulation. We also provide standard HMC sampling speeds as reference.

## Approach

Our modeling framework is motivated by real biological experiments: measurements on systems subject to long term or permanent perturbations, such as gene expression regulation via different promoters, silencing of genes or the stable transfection with variants of the system’s enzymes with altered reactive properties. These types of experiment, among others, provide steady state responses to perturbations. In the following, we assume data is accumulated by observing the steady state under different conditions *u*.

### Model structure

Intracellular processes are often described as biochemical reaction networks, i.e. ODE models with vector fields that are based on chemical reaction kinetics. Here we consider systems of the following form:
1

where the state  describes molecular concentrations,  denotes the model parameters,  is an input vector which describes experimental conditions such as the suppression of certain reactions or the introduction of mutants, and *n*_E_ denotes the number of performed experiments, i.e. the number of different input vectors. We consider constant inputs, the initial conditions *x* (0) = *x*_0_ are assumed to be known and unparameterised and the function *f* shall be smooth. If the model exhibits complex bifurcations, special care has to be taken when dealing with initial conditions and basins of attraction. We do not address these issues here.

In this study we assume that the system converges to a steady state characterised by  for any available input *w*, which is the generic behaviour of such models [12]. Measurements are taken only after the system has reached steady state:
2

where  is the observable model output when input *u*_*j*_ is applied, and  is the steady state of the respective trajectory *φ*(*t*,*x*_0_,*ρ*,*u*_*j*_). We assume linear output functions characterized by the real matrix *C*.

Standard HMC methods and the proposed Newton-Raphson variants differ in the way the steady states are calculated, but are both initialized with a numerical solution to the initial value problem. The parameters *ρ* are always positive in chemical reaction networks. In order to avoid dealing with borders of the positive orthant it is advantageous to sample in logarithmic space *θ* = log (*ρ*). Since we operate on *θ* we will drop *ρ* from all subsequent expressions and consider it implicitly understood that the model functions *f* and *h* will perform the transformation (the same applies to symbolic expressions like ).

Measurements are obscured by noise *δ*_*ij*_, that is, the observed data
3

relates to the measurement model in this way,
4

Gaussian noise is important for a vast amount of biological applications. However, the noise model is not of crucial importance to the sampling algorithms and can be changed as needed [13]. We assume that variances  are available, e.g. from empirical variance estimates of different experimental replicates.

### Bayesian parameter estimation

Equipped with our model and a data set , we define the likelihood function , as a measure of plausibility for each *θ*. In the case of a Gaussian error model, the likelihood given all experimental measurements follows as


We note that we often employ the log-likelihood in many calculations, which we denote . The likelihood of a set of parameters therefore requires the steady state solution  of the ODE model, which is usually calculated by numerical integration of (1) until convergence is achieved. In a Bayesian approach, prior knowledge regarding the plausible values of *p* (*θ*) is incorporated via Bayes’ theorem:
5

where *p* (*θ*) and  are the prior and posterior distributions, respectively. In this work we assume Gaussian priors . The evidence  is a normalization constant that is independent of *θ* and is not needed during Markov chain Monte Carlo sampling, since this term cancels in the calculation of the Metropolis-Hastings acceptance ratio. Expected values of a function *F*(*θ*) with respect to the posterior,
6

may be estimated using Monte Carlo integration given posterior samples,
7

### Riemannian structure of parameter space

Exploration of the posterior distribution for models defined by systems of ODEs is often severely hampered by the strong correlation structure present in the parameter space, which makes it difficult to propose MCMC moves that are far from the current point and accepted with high probability. Recent advances in MCMC attempt to circumvent these issues by utilising the underlying geometric structure induced by the sensitivity of a statistical model [1]. In addition to exploiting gradient information, we can construct MCMC algorithms based on higher order geometric structure by considering the expected Fisher Information, which [14] noted satisfies all the mathematical properties of a metric tensor and hence induces a Riemannian geometry on the parameter space. The use of this geometry allows us to define a measure of distance between sets of parameters in terms of the change in posterior probability, rather than changes in the values of the parameters themselves. In other words, MCMC algorithms based on Riemannian geometry make local moves according to a local coordinate system that automatically adapts based on the local sensitivity of the model, taking small steps in directions of high sensitivity and bigger steps in directions of lower sensitivity, while also taking into account local correlation between pairs of parameters. Such approaches have been shown to work incredibly well on a variety of complex statistical models [1], although computational expense often remains an issue for some classes of models.

The main quantity of interest here is the metric tensor. From the metric tensor, gradient and log-likelihood, we can define a Hamiltonian Monte Carlo algorithm using Riemannian coordinates, rather than the standard Euclidean coordinate system which is typically used. Both of these algorithms are given in the next section, and we refer the reader to [15] for an introductory exposition of Riemannian geometry for MCMC. We may define the (*r*,*s*)th element of a metric *G*(*θ*) based on the posterior distribution in the following manner,


where *G*_*y*_(*θ*) is the expected Fisher Information and *Ξ* is the covariance of the prior. We note that the normalising factor of the likelihood, and prior for that matter, is a constant with respect to *θ* and vanishes in the derivative of its logarithm. One of the advantages of employing Riemannian geometry is that the calculation of *G*_*y*_(*θ*) requires only *first order* sensitivities . For steady state ODE models, we can calculate a general expression for the expected Fisher Information based on a likelihood derived from Gaussian measurement errors with a linear observation model, as defined in (1) and (2):
8

This calculation yields, taking the prior’s contribution into account, the overall metric tensor *G* comprised of an inner product of the sensitivity matrices and the a-priori covariance matrix:
9

where .

## Methods

We have named the algorithms presented in this work using the prefix NR for *Newton-Raphson* and the prefix RM for *Riemannian Manifold*, which will be further explained in this section. When we define the variants of Hamiltonian Monte Carlo, we restrict our description to those aspects of the algorithm that are impacted by the modifications we make. We note that the modifications do not affect the correctness of the algorithms, and we therefore refer the reader to the original HMC and RMHMC publications for proofs of correctness and convergence [1, 11].

The original HMC algorithm is defined on Euclidean space and performs well in practice for probability distributions that do not exhibit strong correlations between parameters. The state of the art RMHMC performs far better for correlated distributions, however the algorithm is computationally more complex to implement. We consider both HMC and RMHMC on our variety of examples, and then present an approach for significantly decreasing the computational cost of implementing both algorithms for steady state systems.

### Hamiltonian Monte Carlo

The Hamiltonian Monte Carlo algorithm can be constructed by introducing an auxiliary variable to extend the state space. We may interpret the auxiliary variable as a momentum variable and the negative log joint distribution may be interpreted as a Hamiltonian system [1, 16]. The main idea is that the induced dynamics of this system may then be used for proposing moves within an MCMC scheme. This is desirable since the dynamics may propose points that are far from the current point and accepted with high probability.

We begin by rewriting the posterior probability as
10

where


The sampling space is then extended by introducing the momentum variable *η*, and we may write the joint distribution as
11

We note that the Hamiltonian function *H* (*η*,*θ*) is simply the negative log joint distribution of our extended state space and can be used to calculate Hamiltonian trajectories according to the differential equations defined in the algorithm below. Given current values for the parameter and momentum variables, we can simulate the Hamiltonian dynamics to propose a new pair of parameter and momentum variables, which are then accepted according to a Metropolis-Hastings ratio to ensure convergence to the correct stationary distribution. The advantage of this approach is that this ratio may be close to 100%, far higher than the typical optimal acceptance ratios for other MCMC algorithms, which are typically between 20*%* and 60*%*. The standard HMC algorithm is given by, 

1. **Transition step**

Starting at *θ* = : *θ* (0) = *θ*_0_, solve the differential equations,
12

for  with initial conditions:
13

where the proposed parameter and momentum variables at time  are given on the right. The above equations are Hamilton’s equations of motion for a particle with momentum *η* in a potential field .

2. **Acceptance step**

Accept *θ*^′^ and *η*^′^ with probability
14

The numerical solution of (12) must be calculated using a symplectic integrator [1], which is exactly time reversible and volume preserving; these properties are required for ensuring convergence to the correct stationary distribution. Since the Hamiltonian laws of motion conserve energy, an analytic solution would provide sample points with perfect acceptance. However the required numerical integrators introduce a small amount of error and typically sample at very high but lower than 100% acceptance.

This standard version of HMC also profits from efficiently calculating the sensitivities and steady states along Hamiltonian trajectories using our approach based on a multivariate Newton-Raphson method, which we explain in Section ‘Efficient calculation of geometry’. We call this version NR-HMC and include it in our performance evaluation.

### Riemannian manifold Hamiltonian Monte Carlo

Hamiltonian Monte Carlo can also be defined using the induced Riemannian geometry, rather than the usual Euclidean geometry. The RMHMC algorithm may be derived in a similar manner to HMC, except now the momentum variable *η* is defined to be a Gaussian distribution with *position specific* covariance matrix, such that the joint distribution follows as,
15

where *Σ* (*θ*) = *G* (*θ*). In other words, the dynamics now take into account the second order sensitivities of the statistical model of interest, since the covariance of the momentum variable is based on the expected Fisher Information at each point.

This Hamiltonian system is however harder to integrate numerically, since its equations of motion are now defined implicitly due to the dependence of the momentum on the position parameter *θ*. The generalised Leapfrog integrator is the simplest such algorithm for this task, however its use may significantly add to the computational expense of simulating proposals, since a much larger number of likelihood evaluations are generally necessary to solve the equations of motion compared to standard HMC [1, 15]. In addition to the state sensitivities, we require the gradients of *G* (*θ*) for solving the Hamiltonian system, which necessitates the calculation of second order sensitivities, denoted from now on by . This provides the motivation for the next subsection, in which we propose a computationally efficient approach to evaluate the required geometric quantities for enabling faster inference in steady state ordinary differential equation models.

### Efficient calculation of geometry

Practical implementation of Hamiltonian Monte Carlo samplers depends critically on the ability to efficiently calculate the required geometric quantities. Here we show how this may be done for steady state systems.

Output sensitivities  (for any given input ) occur in several places in the RMHMC algorithm. For ODE models, they are needed to construct the metric tensor *G* and the gradient of . In the case of steady state data, the sensitivities can be obtained from the steady state condition:
16

We will drop the arguments of  to shorten notation. In (16) we see that the steady state sensitivity is obtained by solving the following linear algebraic equation,
17

where . We denote the solution to (17) as , which is easy to obtain when the Jacobian is invertible^a^. Similarly, we can write the following equation for the second order sensitivity,
18

leading to a linear equation for the second order sensitivity ,
19

Again, the existence of a solution depends on the invertibility of the Jacobian . We note that the same LU-decomposition of *J*_*f*_ can be used for the first and second order sensitivities. Usually all derivatives of *f* appearing in (16) and (19) can be calculated analytically; for large systems, a symbolic calculation package will do the job, e.g. GiNaC, GNU Octave[forge]’s symbolic package or MATLAB’s symbolic toolbox. A particularly convenient way of storing the models and doing the symbolic calculations is VFGEN[17] which provides MATLAB output.

Equipped with these instructions for sensitivity calculations we can easily calculate the metric *G* (*θ*) and the gradient of the log-likelihood for a given value of *θ*,
20

Although HMC algorithms make large transitions across the parameter space, the transitions are constructed using multiple small integration steps of the Hamiltonian dynamics (12). Since these parameter changes in the integration are gradual, the sensitivity can be used directly to estimate the steady state  after each small parameter step *Δ*,
21

For this reason, it is very convenient and efficient to recalculate the steady states using a Newton Raphson method, where we begin with  at the estimate (21),
22

and for any input *w*, (22) is iterated until satisfactory precision is reached, otherwise we proceed exactly as in the original HMC algorithm. We call these variants NR-HMC and NR-RMHMC.

### Sampling efficiency

For the estimation of the auto-correlation we employed the MATLAB script UWerr documented in [18]. By measuring the execution time *t*_E_ of the sampling we can calculate an effective sampling speed, corrected for auto-correlations:
23

where *N* is the sample size and *τ*_int.,*L*_ the integrated auto-correlation length with respect to the estimation of the mean log-likelihood.

Large auto-correlations reduce the effective speed *v*: the sampled points strongly depend on their predecessors and many Markov chain moves are required to obtain independent information about the posterior distribution. A sampling method with increased cost per Markov chain transition might outperform a simple-but-cheap method if the returned points are sufficiently less correlated. The effective speed *v* takes the purpose of sampling into account. When comparing algorithms we also list a relative speed *v*_*r*_, where we normalize each *v*_*i*_ using the lowest observed speed.

## Results and discussion

In this section we apply the Bayesian parameter estimation framework to three examples from literature, which feature the type of data and modeling approach described in Section ‘Methods’. A comparison of the performance of all algorithms is provided in Additional file 1: Figure S1.

All simulations were done in MATLAB. We used the SBPOP Toolbox bundle [19] to store the models and solve the ODE () for RMHMC. The implementation makes use of the symmetry of the second order sensitivity: , and reuses appropriate fluxes, nevertheless the integrator used by the toolbox has to integrate an initial value problem with *n* + *nm* + *nmm* state variables (number of states, number of first order sensitivities, number of second order sensitivities).

The models are stored as symbolic variables for the Newton-Raphson type algorithms, symbolic calculations of the required derivatives of *f* then yield a standard matlab function for the Jacobian and the sensitivities. All linear equations are solved using MATLAB’s backslash operator. We provide MATLAB code for easy reproduction of all of the results in this section.

Since the methods we used are not restricted to the RMHMC algorithm, we also tested the simplified Metropolis-adjusted Langevin algorithm (SMMALA) [1]. We applied the same modifications to SMMALA and measured the relative sampling speed, which can be inspected in Table 1.

**Table 1:** **Effective sampling speed measurements for SMMALA and the modified NR-SMMALA**

Problem size		NR-SMMALA	SMMALA
2×2	*v* in *s* ^-1^	90±2	71±2
	*τ*_int.,*L*_	0.88±0.02	0.88±2
3×6	*v* in *s* ^-1^	62±1	32±1
	*τ*_int.,*L*_	1.20±0.03	1.52±0.04
6×14	*v* in *s* ^-1^	0.30±0.07	0.031±0.006
	*τ*_int.,*L*_	290±67	166±31

### Example: Erk phosphorylation in MAPK signaling

We consider a modification of the model for Erk phosphorylation in the MAPK signaling cascade introduced in [20], who investigated robustness of steady state phosphorylation of Erk with respect to changes in the total Erk amount Erk _T_= *u* :
24

where *x* = ([ pErk],[ ppErk])^T^ are the once and twice phosphorylated modifications of Erk.

According to the conclusions in [20] the robustness of this system with respect to varying Erk _T_ is due to negative feedback from Erk to Raf and MEK; this was not investigated by the authors using an ODE model but directly in experiment and by modifying the steady state solution of the open loop system. To account for this negative feedback we modified the phosphorylation rate of the original model by multiplying it with *u*/(1+*u*). This modification of the model does provide negative feedback, though we do not suggest any specific mechanism or interpretation for it, but rather we aim at illustrating how a modeling hypothesis can be tested. The two different Erk variants can be knocked out individually, but have a very similar function. This enables the experimentalists to reduce the amount of Erk to several intermediate levels. For more details on the biological system we refer to [20].

The normalized data provided in the supplementary material of [20] contains 10 steady state measurements of *x*_2_ obtained with western blots under different perturbation conditions in which Erk _1_ and/or Erk _2_ were knocked down, resulting in different Erk _*T*_ concentrations. The data point belonging to the experiment in which no knockdown was performed serves as control experiment with *u*=1 in normalized units.

Unfortunately these measurements are not acompanied by information about standard deviations in the publication. For the purposes of an illustrative example we suggest that the value *σ*_*ij*_= 0.2 seems reasonable. The corresponding measurement model reads
25

**Figure 1:**
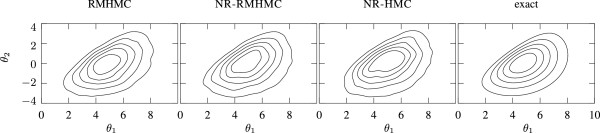
**MAPK posterior comparisons fltr.** (1) posterior inferred from RMHMC sample. (2) Newton Raphson driven RMHMC posterior inferred from sample. (3) posterior inferred from NR-HMC sample (4) exact posterior on a grid. We used a kernel density estimator (*kde*) to infer densities from samples.

We used a normal prior in the logarithmic parameter space which covers several orders of magnitude for both parameters. This example has two parameters, making it convenient to compare the kernel density estimate of the posteriors to an evaluation of the posterior on a regular grid, which removes any sampling related errors. This comparison can be seen in Figure 1. All posteriors look very similar, which indicates proper convergence for all three algorithms.

When looking at Additional file 1: Figure S1, we see that the effective sampling speed of NR-RMHMC is 2.6 times higher than that of the original RMHMC, while NR-HMC is better still.

### Example: insulin dose response

A larger example is provided in [21], in which the authors analyze the insulin pathway in eukaryotic cells (primary human adipocytes). Different models were tested in their ability to describe phosphorylation of the insulin receptor substrate (IRS) after insulin stimulation. The data sets provided in their supplementary material consist of dose response curves, which we interpret as steady state data, as well as dynamic responses, which we disregard here.

**Figure 2:**
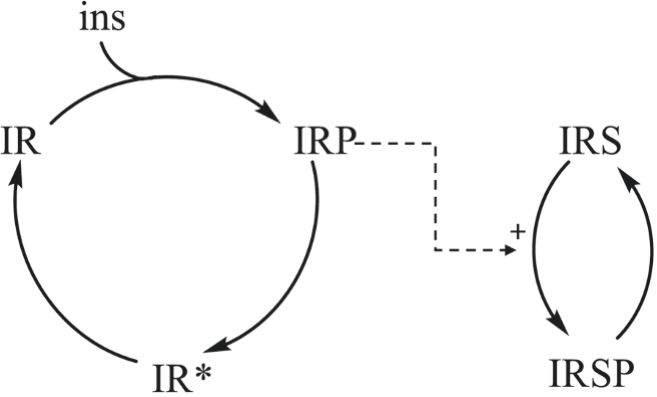
**Interaction graph of the Mma model of** [21] **for IRS phosphorylation after insulin stimulation.**

From the set of models provided, we chose one of the least complex models (shorthand label: Mma) with *m*=6 parameters, which was nevertheless sufficient to fit the steady state data, as well as the best fitting model (Mifa). The interaction graph of Mma is shown in Figure 2. The model comprises 5 molecular species: the insulin receptor (IR and IR ^∗^), phosphorylated IR (IRP), IR substrate (IRS), and phosphorylated IRS (IRSP). These reactants form two groups with built in conservation relations. Since the sums
26

do not change over time, we only require *n*=3 independent state variables to write down the ODE model:


which defines the initial value problem (ivp) for


with measurement model
27

We have used the value for the output parameter *C*_1,3_ reported in the publication and will not estimate it during sampling.

**Figure 3:**
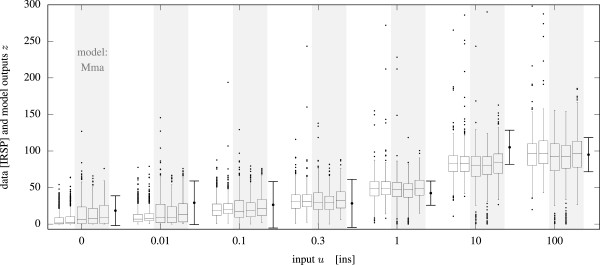
**Comparison of five different fits for the insulin dose resonse data.** The data is shown as error bars (last in each group), the fits are shown in boxplot style in the order: (i) Mifa model and NR-RMHMC sampler, (ii) Mifa model NR-HMC sampler, (iii) Mma model NR-RMHMC sampler, (iv) Mma model NR-HMC sampler, (v) Mma model and unchanged RMHMC sampler with numerical ODE solutions using SBToolBox2[mex]. All algorithms succeeded in fitting the data.

The larger Mifa model, which comprises 6 independent state variables and 14 parameters and is capable of fitting dynamic responses as well, is treated similarly and included as SB model in the supplement.Figure 3 shows a comparison of fits using different models and samplers, illustrated as box plots. The boxes fit the observed experimental data well (error bars). It was not computationally feasible to sample the 14 parameter Mifa model on our desktop machine using the unmodified RMHMC algorithm.

As shown in Additional file 1: Figure S1, the effective speed of NR-RMHMC was about 23 times higher than that of standard RMHMC for the 3×6 Mma model, indicating a significant speed up. The largest observed correlation coefficient of the posterior was *ϱ*_56_(Mma)≈0.8. Since the posterior does not exhibit strong enough correlations between the parameters, the flat (Euclidean) metric used in NR-HMC was of no disadvantage and hence NR-HMC performed best. With the larger Mifa model RMHMC came to its limits, while the modified NR-RMHMC was still able to generate samples in an acceptable time. The use of a Newton-Raphson approach for this model resulted in two orders of magnitude improvement in sampling performance for the NR-HMC algorithm. Although NR-HMC was superior in cases with uninformative priors, the advantage of NR-RMHMC becomes evident in the case of an informative prior. The informative prior was built by assigning smaller variances to some of the parameters *θ*_*i*_ while keeping the rest vague (large variances). See the setup files in the supplement for details.

## Conclusion

We have demonstrated on three real world examples of different sizes how Hamiltonian Monte Carlo methods may be implemented in an efficient manner for the class of steady state dynamical systems. Using sensitivity analysis to track the steady states during each (RM)HMC trajectory calculation in the proposal step leads to a significant improvement in terms of effective sampling speed in all examples. Furthermore, the speed up was even more pronounced for larger problems comprising more parameters.

**Figure 4:**
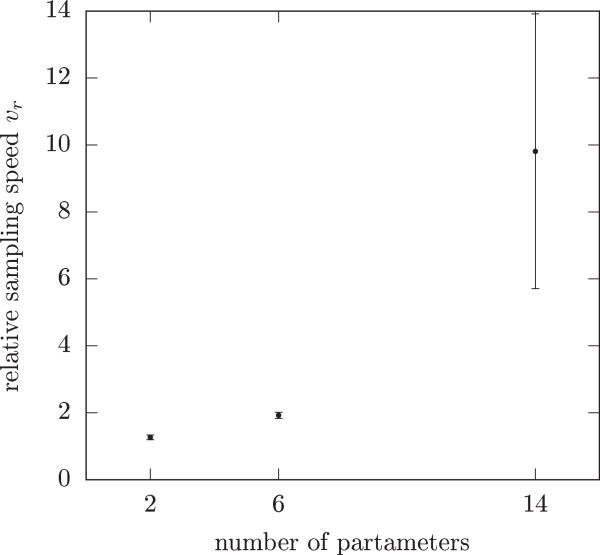
**Relative speed for the Simplified Manifold Metropolis-Adjusted Langevin Algorithm (SMMALA) using the same modifications as in the RMHMC code.** We calculate the sensitivities using systems of linear equations, we use the sensitivities to obtain the metric and the starting point for the Newton Raphson method which is used to calculate steady states. We tested the relative speed of both the modified and unmodified SMMALA on all three example models with uninformative priors. In all cases, the modified version of SMMALA was faster than the reference implementation.

The proposed approach is also applicable to other Riemannian manifold MCMC algorithms like SMMALA. Figure 4 shows that there are significant albeit less dramatic benefits for the introduced techniques for this algorithm as well. Once again, we can use the comparatively inexpensive steady state sensitivity analysis to obtain the metric tensor *G* as well as the Newton-Raphson method for the calculation of steady states to great effect.

There remains the question of whether to employ NR-HMC or the more complicated NR-RMHMC algorithm for performing inference over steady state systems. In most cases, steady state data is not sufficient to uniquely fit the parameters to specific values, and we are unable to know a priori whether the parameters in the system will exhibit strong correlation structure. In practice, one might therefore start with the simpler NR-HMC scheme, and resort to the Riemannian version of it if the need arises. In cases where the prior may be well specified, the differences in scale between different parameters may require NR-RMHMC for improved efficiency, as we observed in the example section.

We conclude that the use of Newton-Raphson methods for calculating the steady states within HMC algorithms is a valuable contribution to the field of numerical parameter estimation for this special class of ODE models and improves scalability of statistical sampling-based approaches for these models in general. The success of this approach motivates further development of HMC algorithms for the more general case of dynamic time series data, which would broaden its utility.

## Endnote

^a^ We note that this is not always the case: whenever conservation relations are present, for example, the Jacaobian is not invertible anywhere. However, in such cases it is sufficient to use these conservation relations to reduce the number of state variables, as we do in the examples.

## Electronic supplementary material

Additional file 1: Figure S1.: Performance analysis for the original RMHMC for ODE models and two steady state data adapted HMC algorithms. (PDF 80 KB)
