# Supplementary material for: Hamiltonian Monte Carlo methods for efficient parameter estimation in steady state dynamical systems
Source: BMC Bioinformatics. 2014 Jul 28;15(1):253. doi: 10.1186/1471-2105-15-253 (PMC4262080; doi:10.1186/1471-2105-15-253)
Supplement: Supplementary file 1 — Additional file 1: Figure S1.: Performance analysis for the original RMHMC for ODE models and two steady state data adapted HMC algorithms. (PDF 80 KB) [file 12859_2014_6684_MOESM1_ESM.pdf]

Figure S1. Performance analysis for the original RMHMC for ODE models and two steady state data adapted HMC algorithms

$p(\theta)$	model	size		RMHMC (Riemannian Manifold)	NR-RMHMC (Riemannian Manifold; Newton Raphson)	NR-HMC (Newton Raphson)	HMC
u	MAPK	2×2	v in s^{-1}	0.312 ± 0.015	0.811 ± 0.040	4.19 ± 0.29	0.682 ± 0.088
			v_r	1	2.60 ± 0.25	13 ± 2	2.19 ± 0.39
			$\tau_{\text{int.},L}$	1.491 ± 0.070	1.633 ± 0.080	3.60 ± 0.24	16 ± 2
u	Mma	3×6	v in s^{-1}	$(5.8 \pm 1.0) \times 10^{-4}$	$(1.35 \pm 0.24) \times 10^{-2}$	0.376 ± 0.065	$(2.46 \pm 0.86) \times 10^{-2}$
			v_r	1	23 ± 8	640 ± 230	42 ± 22
			$\tau_{\text{int.},L}$	18 ± 3	19 ± 3	17 ± 3	42 ± 15
u	Mifa	6×14	v in s^{-1}	$< 9 \times 10^{-6}$	$(1.69 \pm 0.25) \times 10^{-2}$	$(4.92 \pm 0.75) \times 10^{-1}$	$(5.2 \pm 1.3) \times 10^{-3}$
			v_r	1	$> 1930 \pm 290$	$> 55000 \pm 8000$	$> 580 \pm 150$
			$\tau_{\text{int.},L}$	NA	11 ± 2	12 ± 2	42 ± 10
i	Mifa	6×14	v in s^{-1}	NA	$(2.36 \pm 0.12) \times 10^{-1}$	$(1.76 \pm 0.41) \times 10^{-1}$	$(4.5 \pm 1.3) \times 10^{-3}$
			v_r		52 ± 18	39 ± 20	1
			$\tau_{\text{int.},L}$		0.87 ± 0.04	36 ± 8	62 ± 17

The problem size is $n \times m$, where n is the number of state variables and m the number of parameters. The properties listed are the effective sampling speed v, relative speed v_r, and the integrated auto-correlation length $\tau_{\text{int.},L}$. HMC with flat metric (last column) performs less efficient moves through parameter space, which sometimes results in higher auto-correlation. In our examples this efficiency loss is often compensated by lower computational costs. The last example, where we resampled the same 6×14 model with an informative prior on half of the parameters, illustrates the advantages of Riemannian manifold methods over their non-Riemannian counterparts: NR-RMHMC is faster than NR-HMC. The sample sizes N for the various models: (MAPK) $N = 20000$, (Mma) $N = 10000$, (Mifa) $N = 10000$.
